# Pulmonary Alveolar Proteinosis During Intensive Immunosuppressive Treatment for Acute Exacerbation of Interstitial Pneumonia: A Case Report and Literature Review

**DOI:** 10.7759/cureus.74669

**Published:** 2024-11-28

**Authors:** Atsushi Yanagisawa, Hachiro Konaka, Masaki Tanaka, Shoichi Ihara, Isao Tachibana

**Affiliations:** 1 Department of Respiratory Medicine and Clinical Immunology, Nippon Life Hospital, Osaka, JPN; 2 Department of Respiratory Medicine and Clinical Immunology, Osaka University, Osaka, JPN

**Keywords:** acute exacerbation of interstitial pneumonia, immunosuppressive treatment, krebs von den lungen-6, pulmonary alveolar proteinosis, secondary pulmonary alveolar proteinosis

## Abstract

Pulmonary alveolar proteinosis (PAP) is a rare disease characterized by the accumulation of surfactants in the alveoli. It has been suggested that immunosuppressants contribute to the development and exacerbation of PAP. Here, we report the case of a 73-year-old man who developed secondary PAP after intensive immunosuppressive treatment for acute exacerbation of interstitial pneumonia (IP). In this case, despite the improvement of the inflammatory response after immunosuppressive treatment, Krebs von den Lungen-6 (KL-6) continued to increase, leading to the diagnosis of PAP. De-escalation of immunosuppressive treatment improved the PAP, allowing him to be discharged from the hospital. Although KL-6 is a useful marker of IP, when IP appears to be refractory and KL-6 increases despite the improvement of other inflammatory markers, physicians should consider the development of PAP and perform proactive bronchoscopic evaluation.

## Introduction

Pulmonary alveolar proteinosis (PAP) is a rare disease characterized by alveolar accumulation of surfactants due to impaired surfactant clearance by alveolar macrophages [1]. PAP is clinically classified into three distinct forms: autoimmune, secondary, and congenital [1]. Secondary PAP (SPAP) is defined as PAP occurring alongside an underlying condition such as hematological, infectious, autoimmune diseases, or drug toxicities, which test negative for anti-granulocyte-macrophage colony-stimulating factor antibodies (GMAb) [1]. Although the pathogenesis of SPAP remains unclear, it is suggested that the underlying disease causes an abnormal number and function of alveolar macrophages, resulting in impaired surfactant clearance [2]. SPAP is a very rare lung disorder comprising approximately 10% of cases of acquired PAP; however, a certain number of underdiagnosed SPAP cases have not been adequately examined because of the poor general condition caused by the underlying diseases [3]. Here, we report a case of SPAP that developed during the intensive immunosuppressive treatment for refractory interstitial pneumonia (IP).

## Case presentation

**Figure 1:**
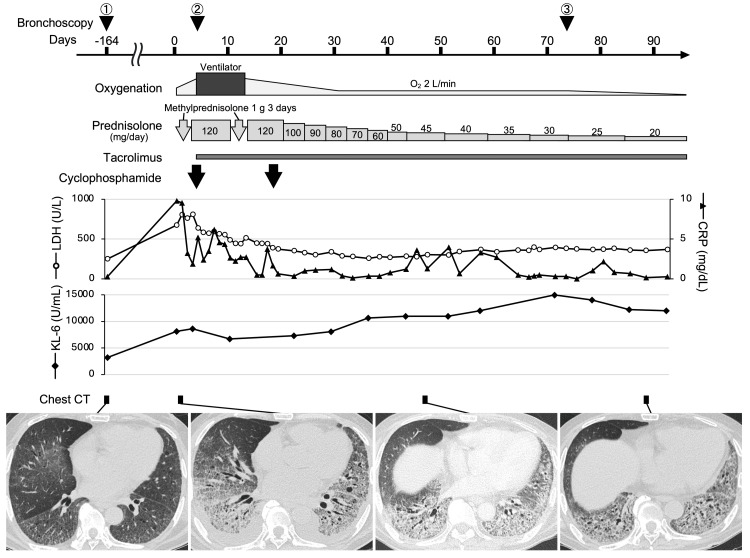
Clinical course. LDH: lactate dehydrogenase; CRP: C-reactive protein; KL-6: Krebs von den Lungen-6; CT: computed tomography; IP: interstitial pneumonia. All days are counted from the day of admission due to acute exacerbation of IP.

A non-smoking 73-year-old man initially presented to our hospital with abnormal shadows that were incidentally detected on chest computed tomography (CT). He had no history of hematological or autoimmune diseases. Although the serum rheumatoid factor was positive at 261 U/mL, other autoantibodies were negative and no clinical signs indicative of autoimmune disease were present. His serum Krebs von den Lungen-6 (KL-6) level was high at 3,192 U/mL. Chest CT showed ground-glass and reticular opacities and peripheral traction bronchiectasis in both his lower lobes but no crazy-paving pattern characteristic of PAP (Figure 1). Bronchoalveolar lavage (BAL) and transbronchial lung biopsy (first bronchoscopy) revealed non-specific interstitial changes but no evidence of PAP. Based on these findings, the patient was diagnosed with IP. A surgical lung biopsy was not performed due to its invasive nature and lack of patient consent. The patient was therefore provisionally diagnosed with unclassifiable idiopathic IPs.

Approximately five months later, the patient was admitted to the hospital with dyspnea. Chest CT on admission showed newly appeared extensive ground-glass opacities. He was diagnosed with acute exacerbation of IP, and immunosuppressive treatment was initiated (Figure 1). On day 4 after admission, his respiratory status deteriorated, necessitating ventilation. BAL performed in the lingular division of the left lung under intubation (second bronchoscopy) showed no turbidity and no evidence of PAP on cytological examination. Intensive immunosuppressive treatment with high-dose steroids, tacrolimus, and cyclophosphamide was administered to control IP progression (Figure 1).

**Figure 2:**
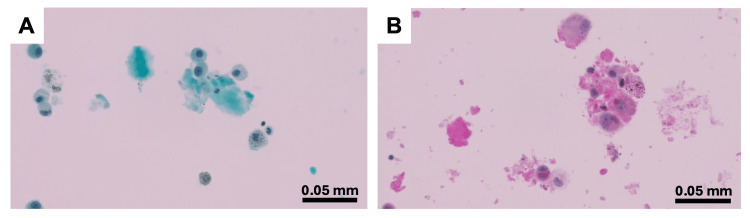
Cytopathological examination of the BAL on day 73. BAL on day 73 (third bronchoscopy) showed abundant extracellular granular material (A, Papanicolaou stain) that was PAS positive. PAS-positive material is also present in alveolar macrophages (B, PAS stain). PAS: periodic acid-Schiff; BAL: bronchoalveolar lavage.

Despite improvement in inflammatory markers such as serum lactate dehydrogenase and C-reactive protein following immunosuppressive treatment, abnormal shadows in both lower lobes remained, and oxygen demand did not improve at 2 L/min. Furthermore, serum KL-6 level, which had decreased slightly shortly after the start of the immunosuppressive treatment, increased to 14,981 U/mL (Figure 1). Bronchoscopy on day 73 (third bronchoscopy) revealed that the BAL from the lingular division of the left lung (the same area examined on day 4) was turbid. This BAL fluid showed characteristic amorphous eosinophilic materials, which were positive for periodic acid-Schiff staining, and foamy macrophages with phagocytosing granular materials, consistent with PAP (Figure 2). Serum anti-GMAb levels were within the normal range, and new-onset SPAP was diagnosed. The culture of the third BAL fluid was negative for bacteria, acid-fast bacilli, and fungi. Therefore, we considered complications of infection to be negative and did not identify any other possible underlying causes of SPAP other than immunosuppressive treatment. Considering the possibility that immunosuppressive treatment exacerbated SPAP, we carefully reduced the dose of steroids while continuing to take tacrolimus. Consequently, oxygenation gradually improved, and oxygen administration was no longer required on day 100. Serum KL-6 also decreased after the third bronchoscopy, and the ground-glass opacities on chest CT also slowly diminished (Figure 1).

## Discussion

Although the etiology of SPAP remains unclear, it is possibly caused by abnormal differentiation and dysfunction of alveolar macrophages. Immunosuppressive drugs can cause SPAP [1,3] and are also associated with progression or exacerbation of PAP [2,4]. In this case, the findings of PAP, which were not observed in the first two BALs prior to immunosuppressive treatment, were confirmed in the third BAL, leading us to suspect that immunosuppressive treatment caused the onset and exacerbation of PAP. Discontinuation of the suspected drug is important in drug-induced PAP [3]. In this case, we carefully reduced the dose of steroids, given concerns about a relapse of IP, and we succeeded in improving the ground-glass opacity in the chest; however, the approach for adjusting immunosuppressive drugs when PAP coexists with an exacerbation of IP for which immunosuppressive treatment is required remains unclear.

Prior to this patient, there have been 13 documented reports on the development of PAP during immunosuppressive treatment for IP (Table 1) [5-16]. Among them, 10 cases (cases 1, 2, 5, 6, 7, 8, 9, 11, 12, and 13) of IP were associated with dermatomyositis. Four cases (cases 1, 4, 7, and 13) of PAP were classified as SPAP, and the others as autoimmune PAP (APAP). Except for case 4, in which SPAP was caused by chronic myeloid leukemia, intensive multidrug immunosuppressive therapy was administered in all cases. Notably, in eight cases (cases 2, 4, 6, 7, 10, 11, 12, and 13), the increase in KL-6 during immunosuppressive treatment was attributed to the exacerbation of IP rather than the onset of PAP, leading to intensified immunosuppressive treatment and subsequent exacerbation of PAP. In our case, it was difficult to decide whether to escalate immunosuppressive treatment alone based on serum biomarkers and radiological findings when serum KL-6 levels were elevated. However, bronchoscopic evaluation allowed a definitive diagnosis of PAP and an appropriate reduction in immunosuppressive treatment. To avoid unnecessary immunosuppression, bronchoscopic evaluation may be recommended in cases of refractory IP with elevated KL-6 levels in contrast to the improvement of inflammation. If PAP is diagnosed during immunosuppressive treatment, physicians should consider that immunosuppressive treatment may exacerbate PAP and titrate immunosuppressive drugs regardless of KL-6 levels if other findings show an improvement in IP.

**Table 1:** Cases of PAP that developed during immunosuppressive treatment for IP. ^#^Values estimated from the line graphs in the figures, although the actual values are not provided in the main text. *Specific drug name is not mentioned in the text. IP: interstitial pneumonia; PAP: pulmonary alveolar proteinosis; KL-6: Krebs von den Lungen-6; MDA-5: melanoma differentiation-associated gene 5; Ab: antibody; DM-ILD: dermatomyositis-associated interstitial lung disease; PSL: prednisolone; CyA: cyclosporine A; IVCY: intravenous cyclophosphamide; ARS: aminoacyl-tRNA synthetase; AZA: azathioprine; CML: chronic myeloid leukemia; TAC: tacrolimus; TFB: tofacitinib.

	Year	Age/sex	Type of IP	Immunosuppressive treatment for IP	Type of PAP	Serum KL-6 level before immunosuppressive treatment	Highest serum KL-6 levels	Whether or not immunosuppressive treatment was intensified when KL-6 was elevated because of the onset of PAP
Case 1 [5]	2018	58, Female	Anti-MDA5 Ab-positive DM-ILD	PSL + CyA + IVCY	Secondary	714 U/mL	15,600 U/mL	
Case 2 [5]	2018	58, Male	Anti-ARS Ab-positive DM-ILD	PSL + CyA + IVCY	Autoimmune	847 U/mL	6,270 U/mL	Intensified
Case 3 [6]	2018	73, Male	Unclassifiable idiopathic IPs	PSL + AZA	Autoimmune	6,429 U/mL	7,900 U/mL^#^	
Case 4 [7]	2019	72, Male	Organizing pneumonia	PSL	Secondary (CML)	100 U/mL^#^	>5,000 U/mL^#^	Intensified
Case 5 [8]	2020	52, Female	Anti-ARS Ab-positive DM-ILD	PSL + CyA	Autoimmune	4,100 U/mL^#^	6,843 U/mL	
Case 6 [9]	2022	66, Male	Anti-ARS Ab-positive DM-ILD	PSL + TAC + IVCY	Autoimmune	10,513 U/mL	>5,000 U/mL	Intensified
Case 7 [10]	2022	48, Male	Anti-MDA5 Ab-positive DM-ILD	PSL + TAC + IVCY + TFB	Secondary	1,000 U/mL^#^	18,683 U/mL	Intensified
Case 8 [11]	2022	61, Male	Anti-ARS Ab-positive DM-ILD	PSL + TAC + IVCY	Autoimmune	1,860 U/mL	5,153 U/mL	
Case 9 [12]	2023	78, Male	DM-ILD (Ab related to DM were negative)	PSL + TAC + immunoglobulin therapy	Autoimmune	-	3,170 U/mL	
Case 10 [13]	2023	73, Female	Fibrotic hypersensitivity pneumonitis	PSL + CyA	Autoimmune	8,758 U/mL	26,974 U/mL	Intensified
Case 11 [14]	2024	50, Female	Anti-MDA5 Ab-positive DM-ILD	PSL + TAC + IVCY	Autoimmune	250 U/mL^#^	5,200 U/mL^#^	Intensified
Case 12 [15]	2024	46, Male	Clinically amyopathic DM-ILD	Corticosteroid + immunosuppressive agent^＊^	Autoimmune	-	6,517 U/mL	Intensified
Case 13 [16]	2024	70, Female	Anti-ARS Ab-positive DM-ILD	PSL + TAC + IVCY	Secondary	1,197 U/mL	4,743 U/mL	Intensified
Our case	2024	73, Male	Unclassifiable idiopathic IPs	PSL + TAC + IVCY	Secondary	3,192 U/mL	14,981 U/mL	

Serum KL-6 levels are generally higher in PAP cases than in other IP cases. It has been reported that the cut-off value for serum KL-6 to differentiate APAP from other IPs is 2,050 U/mL [17], though low KL-6 levels do not necessarily exclude the diagnosis of PAP. Furthermore, it has also been reported that serum KL-6 levels in SPAP are as high as in APAP [18]. In seven of the 14 cases in Table 1, the serum KL-6 levels before the diagnosis of PAP exceeded 2,050 U/mL, an atypically high level for IP. Although IP has not been established as an underlying disease for SPAP, chronic IP can have focal histological features similar to PAP, termed "PAP-like change" [19]. We suspect that these patients may have had a potential predisposition to developing PAP. Further investigations are warranted to elucidate the specific features of IP that may lead to PAP following immunosuppressive therapy.

## Conclusions

Here, we report a case of SPAP that developed during intensive immunosuppressive treatment for acute exacerbation of IP. In cases of refractory IP in which KL-6 increases in contrast to the improvement of inflammation, we should suspect the development of PAP and perform proactive bronchoscopic evaluation.
